# A Systematic Review of Pediatric Abusive Head Trauma: What a Surgeon Needs to Know Before Using a Knife

**DOI:** 10.7759/cureus.78419

**Published:** 2025-02-03

**Authors:** Jitender Chaturvedi, Divakar Goyal, Ritu S, FNU Ruchika, Mohd Altaf Mir

**Affiliations:** 1 Neurosurgery, All India Institute of Medical Sciences, Rishikesh, Rishikesh, IND; 2 Trauma and Emergency, All India Institute of Medical Sciences, Bathinda, Bathinda, IND; 3 Anaesthesia, Fortis Hospital, Mohali, IND; 4 Trauma Surgery and Critical Care, All India Institute of Medical Sciences, Rishikesh, Rishikesh, IND; 5 Neurological Surgery, Johns Hopkins University, Baltimore, USA; 6 Burns and Plastic Surgery, All India Institute of Medical Sciences, Bathinda, Bathinda, IND

**Keywords:** abusive traumatic brain injury, child abuse, child abuse and neglect, child safety, child trauma

## Abstract

Abusive head trauma (AHT) has been defined as an injury to the skull or intracranial contents of a baby or young child (age less than five years) due to inflicted blunt impact and/or forceful shaking. It has a substantial impact, such as blindness and severe developmental delay in the short and long run. We searched PubMed, Cochrane, Embase, and Google Scholar databases for articles published between January 2001 and December 2021. Two independent reviewers extracted the data from the included studies. The Joanna Briggs Institute (JBI) critical appraisal tool was used to assess the bias in the studies included. A total of 58 studies were included for review. Out of them, 20 studies looked at the risk factors, 28 studies addressed the clinical features, 28 publications described extracranial injuries, 26 papers discussed radiological procedures and various abnormalities, and 31 studies discussed outcomes. We hope to share with clinicians and surgeons the information learned by conducting this review about clinical aspects, patterns of injuries to extra-cranial systems among AHT, radiographic technologies, and upcoming advancements in radiological aspects and overall outcomes of AHT.

## Introduction and background

Child abuse has been classified into four categories: physical abuse, sexual abuse, emotional/psychological abuse, and neglect [1]. Any of the body organs, including the brain, are predisposed to be harmed secondary to physical abuse. Although such injuries were once known as Shaken baby syndrome, the terminology has long been changed to abusive head trauma (AHT). The Centers for Disease Control and Prevention (CDC) describe AHT as "an injury to the skull or intracranial contents of a baby or young child (less than 5 years of age) due to inflicted blunt impact and/or forceful shaking" [2]. AHT has substantial impacts, such as blindness and severe developmental delay in both short and long terms. Mortality is particularly significant in children less than one year of age [3]. t is difficult to differentiate between accidental and non-incidental head trauma based on the clinical findings alone, which are often non-specific. The clinical features are not precise and unique to distinguish AHT from accidental trauma, making it essential to obtain a thorough history of the mechanism of injury, family history, and socioeconomic status to identify risk factors. A comprehensive examination, including clinical and radiological assessments, is needed to identify and improve such patients' outcomes. Before using a knife, knowledge of AHT comes in handy to prevent further trauma to the victim.

In this review, we aim to learn more about the risk factors, clinical aspects, patterns of injuries to extracranial systems among AHT, radiographic technologies, upcoming advancements in radiological aspects, and overall outcomes of AHT. Through this study, we expect a neurosurgeon, reconstructive surgeon, or trauma surgeon to understand this clinical condition better and be confident enough to suspect, diagnose, and manage such patients effectively.

We addressed our research question by reviewing and analyzing relevant publications using the criteria.

## Review

**Figure 1:**
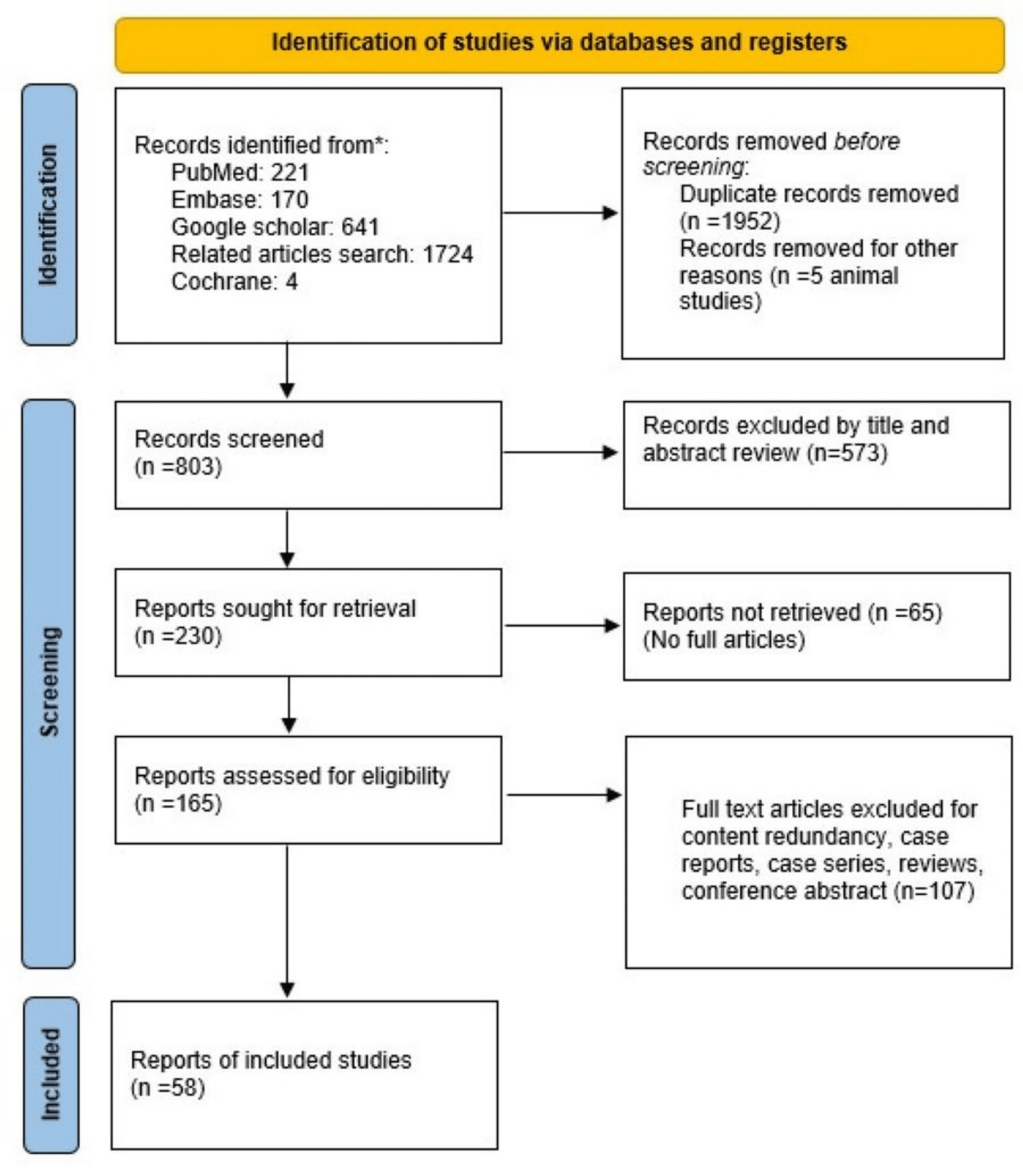
PRISMA flow diagram of study PRISMA: Preferred Reporting Items for Systematic Reviews and Meta-Analysis

The current systematic review was conducted according to Preferred Reporting Items for Systematic Reviews and Meta-Analysis (PRISMA) (Figure 1).

A prior protocol for this review was registered with PROSPERO (no. CRD42022307827).

Literature search strategy

An electronic search was conducted for studies published between January 2001 and December 2021 in the PubMed, Embase, Google Scholar, and Cochrane databases, followed by a hand search. Search phrases were combined in various ways to search the databases. Two independent researchers conducted a literature search and gathered all pertinent publications. Another researcher reviewed all the selected abstracts.

After combining the search phrases "head trauma," "traumatic brain damage," "pediatric," "abusive," "pediatric abusive head injury," and "pediatric abusive head trauma," a total of 2,760 articles were found in the databases. After deleting duplicates and animal studies and studies with titles and abstracts only, a total of 230 articles were studied, of which 65 were excluded due to the non-availability of full articles. After removing studies with foreign languages, case studies, case series, systematic reviews, editorials, and conference papers, we were left with 58 articles written entirely in English.

The final papers, 558 in all, were thoroughly evaluated by two authors with cross-checking by a third reviewer and were divided into five categories: clinical features, risk factors, extracranial injury, radiology, and outcomes are all things to consider.

Assessment of Risk of Bias in Individual Studies

Joanna Briggs Institute's (JBI) critical appraisal tool was used to assess the bias in the studies included. Two authors evaluated each study independently, and differences were resolved by discussion with the corresponding author. The studies were broadly classified into cohort, case-control, analytical cross-sectional, and prevalence studies (Table 1).

**Table 1:** Summary of the studies describing the type of study

S. No.	Author/year	Type of study	
1	Hettler et al., 2003 [4]	Retrospective	Case control
2	Keenan et al., 2003 [5]	Prospective	Cohort
3	Keenan et al., 2004 [6]	Retrospective	Analytical cross-sectional
4	Starling et al., 2004 [7]	Prospective	Cohort
5	Shah et al., 2005 [8]	Retrospective	Cohort
6	Sills et al., 2005 [9]	Retrospective	Cohort
7	Talvik et al., 2006 [10]	Retro+prospective	Cohort
8	Trench et al., 2007 [11]	Retrospective	Cohort
9	Leventhal et al., 2010 [12]	Retrospective	Cohort
10	Díaz-Olavarriea et al., 2011 [13]	Retrospective	Prevalence
11	Greiner et al., 2012 [14]	Retrospective	Cohort
12	Parks et al., 2012 [15]	Retrospective	Cohort
13	Hasbani et al., 2013 [16]	Retrospective	Cohort
14	Babbitt et al., 2013 [17]	Retrospective	Case control
15	Parrish et al., 2013 [18]	Retrospective	Cohort
16	Niederkrotenthaler et al., 2013 [19]	Retrospective	Analytical cross-sectional
17	Kadom et al., 2013 [20]	Retrospective	Cohort
18	Bradford et al., 2013 [21]	Retrospective	Prevalence
19	Roach et al., 2014 [22]	Retrospective	Analytical cross-sectional
20	Choudhary et al., 2014 [23]	Retrospective	Analytical cross-sectional
21	Feldman et al., 2015 [24]	Retrospective	Analytical cross-sectional
22	Westrick et al., 2015 [25]	Retrospective	Cohort
23	Burkhart et al., 2015 [26]	Retrospective	Case control
24	Tung et al., 2015 [27]	Retrospective	Cohort
25	Buttram et al., 2015 [28]	Retrospective	Cohort
26	Vadivelu et al., 2016 [29]	Retrospective	Cohort
27	Khan et al., 2017 [30]	Retrospective	Prevalence
28	Gencturk et al., 2017 [31]	Retrospective	Analytical cross-sectional
29	Kralik et al., 2017 [32]	Prospective	Cohort
30	Morgan et al., 2018 [33]	Retrospective	Analytical cross-sectional
31	Dingman et al., 2018 [34]	Retrospective	Cohort
32	Pierre-Kahn et al., 2018 [35]	Prospective	Cohort
33	Ronning et al., 2018 [36]	Retrospective	Cohort
34	Orru et al., 2018 [37]	Retrospective	Cohort
35	Babl et al., 2018 [38]	Prospective	Cohort
36	Henry et al., 2018 [39]	Retrospective	Cohort
37	Kralik et al., 2018 [40]	Retrospective	Cohort
38	Oh et al., 2019 [41]	Retrospective	Cohort
39	Babl et al., 2019 [42]	Prospective	Cohort
40	Emrick et al., 2019 [43]	Retrospective	Prevalence
41	Lovett et al., 2019 [44]	Retrospective analysis of prospective data	Analytical cross-sectional
42	Wright et al., 2019 [45]	Retrospective	Cohort
43	Thamburaj et al., 2019 [46]	Retrospective	Cohort
44	Sidpra et al., 2020 [47]	Retrospective	Cohort
45	Burns et al., 2020 [48]	Retrospective	Cohort
46	Even et al., 2020 [49]	Retrospective	Analytical cross-sectional
47	Thiblin et al., 2020 [50]	Retrospective	Cohort
48	Sayrs LW et al., 2020 [51]	Retrospective	Cohort
49	Rebbe et al., 2020 [52]	Retrospective	Cohort
50	Hymel et al., 2020 [53]	Prospective	Analytical cross-sectional
51	Henry et al., 2020 [54]	Retrospective, stratified, random systematic sample	Prevalence
52	Rosenfeld et al., 2020 [55]	Retrospective	Cohort
53	Rabbitt et al., 2020 [56]	Retrospective	Cohort
54	Lee et al., 2020 [57]	Retrospective	Cohort
55	Notrica et al., 2021 [58]	Retrospective	Cohort
56	Kriss et al., 2021 [59]	Retrospective	Analytical cross-sectional
57	Manfield et al., 2021 [60]	Retrospective	Cohort
58	Theodorou et al., 2021 [61]	Retrospective	Cohort

They were further divided into subcategories to assess the type of bias in each study. The bias details have been summarized in (Tables 2-5). Table 2 presents the summary of the studies describing various biases in cohort studies.

**Table 2:** Summary of the studies describing various bias in cohort studies

Author/Year	1. Were the two groups similar and recruited from the same population?	2. Were the exposures measured similarly to assign people to both exposed and unexposed groups?	3. Was the exposure measured in a valid and reliable way?	4. Were confounding factors identified?	5. Were strategies to deal with confounding factors stated?	6. Were the groups/participants free of the outcome at the start of the study (or at the moment of exposure)?	7. Were the outcomes measured in a valid and reliable way?	8. Was the follow-up time reported sufficient to be long enough for outcomes to occur?	9. Was the follow-up complete, and if not, were the reasons for loss to follow-up described and explored?	10. Were strategies to address incomplete follow-up utilized?	11. Was appropriate statistical analysis used?
Keenan et al., 2003 [5]	Yes	Yes	Yes	Yes	Yes	Yes	Yes	Yes	Yes	Not applicable	Yes
Starling et al., 2004 [7]	Yes	Yes	Yes	Yes	Yes	Yes	Yes	Yes	Yes	Not applicable	Yes
Shah et al., 2005 [8]	Yes	Yes	Yes	Yes	Yes	Yes	Yes	Yes	Yes	Not applicable	Yes
Sills et al., 2005 [9]	Yes	Yes	Yes	No	No	Yes	Yes	Unclear	Unclear	Not applicable	Yes
Talvik et al., 2006 [10]	Yes	Yes	Yes	Yes	Yes	Yes	Yes	Yes	Yes	Not applicable	Yes
Trench et al., 2007 [11]	Yes	Yes	Yes	Yes	Yes	Yes	Yes	Unclear	Unclear	Not applicable	Yes
Leventhal et al., 2010 [12]	Yes	Yes	Yes	Yes	Yes	Yes	Yes	Unclear	Unclear	Not applicable	Yes
Greiner et al., 2012 [14]	Yes	Yes	Yes	Yes	Yes	Yes	Yes	Unclear	Unclear	Not applicable	Yes
Parks et al., 2012 [15]	Yes	Yes	Yes	Yes	Yes	Yes	Yes	Yes	Yes	Not applicable	Yes
Hasbani et al., 2013 [16]	Yes	Yes	Yes	No	No	Yes	Yes	Yes	Yes	Not applicable	Yes
Parrish et al., 2013 [18]	Yes	Yes	Yes	Yes	Yes	Yes	Yes	Yes	Yes	Not applicable	Yes
Kadom et al., 2013 [20]	Yes	Yes	Yes	Yes	Yes	Yes	Yes	Unclear	Unclear	Not applicable	Yes
Westrick et al., 2015 [25]	Yes	Yes	Yes	Yes	Yes	Yes	Yes	Yes	Yes	Not applicable	Yes
Tung et al., 2015 [27]	Yes	Yes	Yes	No	No	Yes	Yes	Yes	Yes	Not applicable	Yes
Buttram et al., 2015 [28]	Yes	Yes	Yes	No	No	Yes	Yes	Unclear	Unclear	Not applicable	Yes
Vadivelu et al., 2016 [29]	Yes	Yes	Yes	Yes	Yes	Yes	Yes	Yes	Yes	Not applicable	Yes
Kralik et al., 2017 [32]	Yes	Yes	Yes	Yes	Yes	Yes	Yes	Yes	Yes	Not applicable	Yes
Dingman et al., 2018 [34]	Yes	Yes	Yes	Yes	Yes	Yes	Yes	Yes	Yes	Not applicable	Yes
Kahn et al., 2018 [35]	Yes	Yes	Yes	Yes	Yes	Yes	Yes	Yes	Yes	Not applicable	Yes
Ronning et al., 2018 [36]	Yes	Yes	Yes	Yes	Yes	Yes	Yes	Unclear	Unclear	Not applicable	Yes
Orru et al., 2018 [37]	Yes	Yes	Yes	Yes	Yes	Yes	Yes	Unclear	Unclear	Not applicable	Yes
Babl et al., 2018 [38]	Yes	Yes	Yes	Yes	Yes	Yes	Yes	Unclear	Unclear	Not applicable	Yes
Henry et al., 2018 [39]	Yes	Yes	Yes	Yes	Yes	Yes	Yes	Unclear	Unclear	Not applicable	Yes
Kralik et al., 2018 [40]	Yes	Yes	Yes	No	No	Yes	Yes	Yes	Yes	Not applicable	Yes
Oh et al., 2019 [41]	Yes	Yes	Yes	No	No	Yes	Yes	Yes	Yes	Not applicable	Yes
Babl et al., 2019 [42]	Yes	Yes	Yes	No	No	Yes	Yes	Yes	Yes	Not applicable	Yes
Wright et al., 2019 [45]	Yes	Yes	Yes	No	No	Yes	Yes	Yes	Yes	Not applicable	Yes
Thamburaj et al., 2019 [46]	Yes	Yes	Yes	Yes	Yes	Yes	Yes	Yes	Yes	Not applicable	Yes
Sidpra et al., 2020 [47]	Yes	Yes	Yes	Yes	Yes	Yes	Yes	Yes	Yes	Not applicable	Yes
Burns et al., 2020 [48]	Yes	Yes	Yes	Yes	Yes	Yes	Yes	Yes	Yes	Not applicable	Yes
Thiblin et al., 2020 [50]	Yes	Yes	Yes	Yes	Yes	Yes	Yes	Yes	Yes	Not applicable	Yes
Sayrs et al., 2020 [51]	Yes	Yes	Yes	Yes	Yes	Yes	Yes	Yes	Yes	Not applicable	Yes
Rebbe et al., 2020 [52]	Yes	Yes	Yes	No	No	Yes	Yes	Yes	Yes	Not applicable	Yes
Rosenfeld et al., 2020 [55]	Yes	Yes	Yes	Yes	Yes	Yes	Yes	Yes	Yes	Not applicable	Yes
Rabbitt et al., 2020 [56]	Yes	Yes	Yes	Yes	Yes	Yes	Yes	Yes	Yes	Not applicable	Yes
Lee et al., 2020 [57]	Yes	Yes	Yes	Yes	Yes	Yes	Yes	Yes	Yes	Not applicable	Yes
Notrica et al., 2021 [58]	Yes	Yes	Yes	No	No	Yes	Yes	Unclear	Unclear	Not applicable	Yes
Manfield et al., 2021 [60]	Yes	Yes	Yes	No	No	Yes	Yes	Yes	Yes	Not applicable	Yes
Theodorou et al., 2021 [61]	Yes	Yes	Yes	Yes	Yes	Yes	Yes	Yes	Yes	Not applicable	Yes

Table 3 presents the summary of the studies describing various biases in case-control studies.

**Table 3:** Summary of the studies describing various bias in case-control studies

Author/Year	1. Were the groups comparable other than the presence of disease in cases or the absence of disease in controls?	2. Were cases and controls matched appropriately?	3. Were the same criteria used for the identification of cases and controls?	4. Was exposure measured in a standard, valid and reliable way?	5. Was exposure measured in the same way for cases and controls?	6. Were confounding factors identified?	7. Were strategies to deal with confounding factors stated?	8. Were outcomes assessed in a standard, valid and reliable way for cases and controls?	9. Was the exposure period of interest long enough to be meaningful?	10. Was appropriate statistical analysis used?
Hettler et al., 2003 [4]	Yes	Yes	Yes	Yes	Yes	Yes	Yes	Yes	Yes	Yes
Babbitt et al., 2013 [17]	Yes	Yes	Yes	Yes	Yes	Not applicable	Unclear	Yes	Yes	Yes
Burkhart et al., 2015 [26]	Yes	Yes	Yes	Yes	Yes	Not applicable	Yes	Yes	Yes	Yes

Table 4 presents the summary of the studies describing various biases in analytical cross-sectional studies.

**Table 4:** Summary of the studies describing various bias in analytical cross-sectional studies

Author/Year	1. Were the criteria for inclusion in the sample clearly defined?	2. Were the study subjects and the setting described in detail?	3. Was the exposure measured in a valid and reliable way?	4. Were objective, standard criteria used for measurement of the condition?	5. Were confounding factors identified?	6. Were strategies to deal with confounding factors stated?	7. Were the outcomes measured in a valid and reliable way?	8. Was appropriate statistical analysis used?
Keenan et al., 2004 [6]	Yes	Yes	Yes	Yes	No	No	Yes	Yes
Niederkrotenthaler et al., 2013 [19]	Yes	Yes	Yes	Yes	No	No	Yes	Yes
Roach et al., 2014 [22]	Yes	Yes	Yes	Yes	No	No	Yes	Yes
Choudhary et al., 2014 [23]	Yes	Yes	Yes	Yes	No	No	Yes	Yes
Feldman et al., 2015 [24]	Yes	Yes	Yes	Yes	No	No	Yes	Yes
Gencturk et al., 2017 [31]	Yes	Yes	Yes	Yes	No	No	Yes	Yes
Morgan et al., 2018 [33]	Yes	Yes	Yes	Yes	No	No	Yes	Yes
Lovett et al., 2019 [44]	Yes	Yes	Yes	Yes	No	No	Yes	Yes
Even et al., 2020 [49]	Yes	Yes	Yes	Yes	No	No	Yes	Yes
Hymel et al., 2020 [53]	Yes	Yes	Yes	Yes	No	No	Yes	Yes
Kriss et al., 2021 [59]	Yes	Yes	Yes	Yes	No	No	Yes	Yes

Table 5 presents the summary of the studies describing various biases in prevalence studies.

**Table 5:** Summary of the studies describing various biases in prevalence studies

Author/Year	1. Was the sample frame appropriate to address the target population?	2. Were study participants sampled in an appropriate way?	3. Was the sample size adequate?	4. Were the study subjects and the setting described in detail?	5. Was the data not applicable conducted with sufficient coverage of the identified sample?	6. Were valid methods used for the identification of the condition?	7. Was the condition measured in a standard, reliable way for all participants?	8. Was appropriate statistical analysis used?	9. Was the response rate adequate, and if not, was the low response rate Not applied appropriately?
Díaz-Olavarriea et al., 2011 [13]	Yes	Yes	Yes	Yes	Yes	Yes	Yes	Yes	Not applicable
Bradford et al., 2013 [21]	Yes	Yes	Yes	Yes	Yes	Yes	Yes	Yes	Not applicable
Khan et al., 2017 [30]	Yes	Yes	Yes	Yes	Yes	Yes	Yes	Yes	Not applicable
Emrick et al., 2019 [43]	Yes	Yes	Yes	Yes	Yes	Yes	Yes	Yes	Not applicable
Henry et al., 2020 [54]	Yes	Yes	Yes	Yes	Yes	Yes	Yes	Yes	Not applicable

Data Extraction

Two reviewers independently extracted and summarised the data from the 58 included publications in a table under the following headings: 1) name of the author, 2) year of publication, 3) country where the study was performed, 4) study design, 5) number of AHT children, 6) patients' age, 7) pertinent findings, and 8) the conclusion. Another reviewer double-checked the tables before writing the review to ensure the results were accurate. Due to the inclusion of observational studies and lack of homogeneous data in the literature, a systematic review was planned and conducted.

Results

A total of 58 studies were included for review, which looked at risk factors, clinical features, extracranial injuries, radiological investigations, and their findings, along with the overall outcomes of AHT victims. Twenty of the 57 studies looked at the risk variables for AHT, which were classified into four categories: perpetrator history and type, paternal characteristics, child characteristics, and other/admission characteristics. The clinical features of these patients were addressed in 28 research studies, and the extracranial injuries were discussed in 28 publications. Twenty-six papers discussed radiological procedures and various abnormalities, and 31 studies discussed outcomes. Results from all these studies are summarised in Tables 6-7. 

**Table 6:** Summary of the studies describing various clinical and imaging characteristics with various risk factors and outcomes

S. No.	Author/Year	Male	No MOI	Seizures	Retinal Haemorrhage	Acute SDH	Risk Factors	Clinical Features	Extra-Cranial injuries	Imaging	Outcomes
1	Hettler et al., 2003 [4]	27 (55.1%)	34 (69.4%)	17 (34.7%)	39/49	43 (87.8%)	Yes	Yes	Yes	Yes	Yes
2	Keenan et al., 2003 [5]	50 (62.5%)		-	-	-	Yes	-	-	-	-
3	Keenan et al., 2004 [6]		51 (63.8%)	10 (12.5%)	61 (76.3%)	75 (93.8%)		Yes	Yes	-	Yes
4	Starling et al., 2004 [7]	53 (65%)		25 (30.86%)	67 (83%)	73 (90%)	Yes	Yes	-	-	-
5	Shah et al., 2005 [8]	32 (63%)		-	-	12 (23.6%)		Yes	Yes	Yes	Yes
6	Sills et al., 2005 [9]	201 (59.1%)	-	-	-	-	Yes	-	-	-	Yes
7	Talvik et al., 2006 [10]	20 (77%)		13 (50%)	16 (61.5%)	20 (26.9%)	Yes	Yes	Yes	Yes	-
8	Trench et al., 2007 [11]	13 (65%)	-	9 (45%)	11 (57.8%)	20 (100%)	-	Yes	-	Yes	Yes
9	Leventhal et al., 2010 [12]	-	-	-	-	-	Yes	-	Yes	-	-
10	Díaz-Olavarriea et al., 2011 [13]	5 (38%)		8 (62%)	8 (62%)	7 (54%)	Yes	Yes	Yes		Yes
11	Greiner et al., 2012 [14]			34 (48%)				Yes			Yes
12	Parks et al., 2012 [15]	450 (57.7%)	-	-	-	-	-	-	-	-	Yes
13	Hasbani et al., 2013 [16]	16 (50%)		4 (12.5%)				Yes	Yes		
14	Babbitt et al., 2013 [17]	16 (57%)		10 (35.7%)	16 (57%)	26 (92.9%)		Yes	Yes		
15	Parrish et al., 2013 [18]	20 (44%)		-	-	-	Yes	-	-	-	-
16	Niederkrotenthaler et al., 2013 [19]	4583 (60.28%)	-	-	-	-	Yes	-	-	-	Yes
17	Kadom et al., 2013 [20]	-	-	-	-	-	-	-	-	Yes	-
18	Bradford et al., 2013 [21]	-	-	-	-	97 (92%)	-	-	-	Yes	-
19	Roach et al., 2014 [22]	365 (63%)		-	-	443 (76%)		Yes	Yes	Yes	Yes
20	Choudhary et al.,2014 [23]	43 (64%)	-	-	-	54 (81%)	-	-	Yes	Yes	-
21	Feldman et al., 2015 [24]	257 (67% of patients having SDH)		187 (40.7%)	203 (65% of patients with SDH)	291 (63%)	Yes	Yes		Yes	Yes
22	Westrick et al., 2015 [25]	80 (57.6%)			79 (56.8%)	110 (79%)	Yes	Yes	Yes	Yes	Yes
23	Burkhart et al., 2015 [26]	60 (58%)		26 (25%)	103 (100%)	76 (74%)		Yes	Yes	Yes	Yes
24	Tung et al., 2015 [27]	4 (44.4%)	-	-	-	9 (100%)	-	-	-	Yes	-
25	Buttram et al., 2015 [28]	65 (61.9%)	-	-	-	29 (78.3%)	-	-	-	Yes	-
26	Vadivelu et al., 2016 [29]	-	-	-	-	14 (50%)	-	-	-	Yes	Yes
27	Khan et al., 2017 [30]	48 (61%)		-	-	68 (86%)	Yes	Yes		Yes	Yes
28	Gencturk et al., 2017 [31]	-	-	-	7 (44%)	13 (81%)	-	-	Yes	Yes	Yes
29	Kralik et al., 2017 [32]	16 (66.7%)	-	-	-	11 (46%)	-	-	-	Yes	-
30	Morgan et al., 2018 [33]	16 (53.3%)		13 (43%)	19 (63.3%)	29 (97%)		Yes	Yes	Yes	Yes
31	Dingman et al., 2018 [34]	38 (65.5%)		27 (46.6%)	44 (75.8%)	56 (98.2%)	Yes	Yes	Yes	Yes	Yes
32	Kahn et al., 2018 [35]	165(71.4%)		132 (57%)	153 (66.2%)	224 (100%)	-	-	Yes	Yes	Yes
33	Ronning et al., 2018 [36]	57 (57.6%) of 99.	57 (57.6%) of 99	-	62 (62.6%) of 99	-	-	-	-	Yes	-
34	Orru et al., 2018 [37]	37 (65%)	-	-	-	43 (75.4%)	-	-	-	Yes	Yes
35	Babl et al., 2018 [38]	249 (66.9%)		-	-	-	Yes	Yes		Yes	
36	Henry et al., 2018 [39]	143 (61.2%)		-	-	203 (87.2%)	-	-	Yes	Yes	-
37	Kralik et al., 2018 [40]	14 (41.2%)		-	-	-	-	-	-	Yes	-
38	Oh et al., 2019 [41]	150 (55.6%)	-	47 (17.4%)	-	-	-	Yes	Yes	-	Yes
39	Babl et al., 2019 [42]	15 (65.2%)	19 (82.7%)	4 (17.4 %)			Yes	Yes		Yes	Yes
40	Emrick et al., 2019 [43]	67 (55.8%)	78 (65%)	50 (43.5%)	81 (78.3%)	106 (88.7%)	Yes	Yes	Yes	Yes	Yes
41	Lovett et al., 2019 [44]	12 (80%)		11 (73.3%)	3 (20%)	13 (87%)		Yes	Yes	Yes	Yes
42	Wright et al., 2019 [45]	-	-	-	-	85 (100%)	-	-	-	Yes	Yes
43	Thamburaj et al., 2019 [46]	-	-	-	18 (85.7%)	-	-	-	Yes	Yes	-
44	Sidpra et al., 2020 [47]	10 (58.8%)			8 (47.1%)	14 (82.35%)	-	Yes	Yes	Yes	-
45	Burns et al., 2020 [48]	135 (55.3%)	30 (12.3%)	32 (13.1%)	53 (21.7%)		Yes	Yes	Yes		Yes
46	Even et al., 2020 [49]	119 (56.1%)				67 (89.3%)	-	Yes	Yes	Yes	Yes
47	Thiblin et al., 2020 [50]	15 (42%)		1/36	2/36	2 (6%)	Yes	Yes	Yes	Yes	-
48	Sayrs et al., 2020 [51]	131 (54.6%)	109 (45.4%)	53 (10.7%)		104 (33.7%)	Yes	Yes	Yes	Yes	
49	Rebbe et al., 2020 [52]	199 (56.2%)	-	-	-	-	Yes	-	-	-	Yes
50	Hymel et al., 2020 [53]	-	79/160	-	-	-	Yes	-	-	-	-
51	Henry et al., 2020 [54]	88 (51.8%)	-	-	-	-	-	-	Yes	Yes	
52	Rosenfeld et al., 2020 [55]	(59%) of total NAT (i.e., 19,149)	-	-	-	-	-	-	Yes	-	Yes
53	Rabbitt et al., 2020 [56]	-	-	-	-	-	-	-	Yes	Yes	-
54	Lee et al., 2020 [57]	40 (51.3%)	-	-	52 (66.7%)	56 (71.8%)	-	-	-	-	Yes
55	Notrica et al., 2021 [58]	136 (56.2%)	101 (41.7%)	54 (10.9%)		107 (34.2%)	Yes	Yes	Yes	Yes	Yes
56	Kriss et al., 2021 [59]	28 (59.6%)	-	-	-	-	-	-	-	Yes	-
57	Manfield et al., 2021 [60]	31 (56.36%)	-	-	-	-	-	-	-	-	Yes
58	Theodorou et al., 2021 [61]	4352 (60.9%)	-	-	-	-	-	-	-	-	Yes

**Table 7:** Summary of the studies describing various effects of the studies

S. No.	Author/Year	Country	Type of study	n	Age	Results	Conclusion	
1	Hettler et al., 2003 [4]	Boston	Retrospective	49	<3 years	No history of trauma has a specificity of 97% with a PPV of 92% for abuse. And this value reached 100% if the child has persistent abnormal neurological deterioration in 25 cases (51.0%).	Low impact or history of no mechanism of injury along with persistent abnormal neurological status in children with traumatic brain injury should be suspicious of child abuse.	
2	Keenan et al., 2003 [5]	North Carolina	Prospective	80	<2 years	The young age of the mother, low educational status, and premature, as well as multiple births, carried a high risk of child abuse. Military background or rural/extended family too carried a risk.	Preventative strategies are required as child abuse is related to family socioeconomic background and many cases are left unreported.	
3	Keenan et al., 2004 [6]	North Carolina	Retrospective	80	<2 years	SDH and extracranial injuries remained common. However, skeletal surveys and ophthalmoscopy can miss the diagnosis in around 10% of inflicted TBI cases.	Inflicted TBI children are more symptomatic as compared to non-inflicted ones. Skeletal survey and ophthalmoscopy are not sufficient for diagnosis so CT and MRI should be done.	
4	Starling et al., 2004 [7]	USA	Prospective	80	<2 years	SDH was most common followed by retinal hemorrhage, skull fractures, and scalp swelling.	Symptoms are produced immediately in inflicted TBI and the incidence does not change with the mechanism of injury i.e., shaking or impact.	
5	Shah et al., 2005 [8]	USA	Retrospective	51	Mean age: 18 months (range: 1–36 months	39% of patients had skull fractures, while 24% of patients had SDH. SDH was mainly due to focal impact. Among extracranial injuries, 20% were thoracic injuries.	Mechanism of injury should be evaluated on the basis of the child’s clinical features as the type of brain injuries can be the same in both inflicted as well as motor vehicle collisions.	
6	Sills et al., 2005 [9]	Colorado	Retrospective	340	<3 years	Among child abuse, 93 % were due to the top perpetrator of the abuse; with the father, stepfather, or mother’s boyfriend being most common, i.e., 28%.	Intentional TBI is associated with poor outcomes in terms of high mortality rates, both prehospital and in-hospital, and thus preventative strategies are needed.	
7	Talvik et al., 2006 [10]	Estonia	Retro+ prospective	26	<1 year	Seizures are the most common clinical feature and retinal hemorrhage was the most common extracranial injury. Bruises in different regions were also very common.	Child abuse is common among TBI patients, so a history of parents’ behavior towards children as well as special attention towards clinical features and imaging is of paramount importance.	
8	Trench et al., 2007 [11]	Spain	Retrospective	10	<2 years	Seizures and head injuries were the most common reasons for consultation in SDH patients. 11 patients had retinal hemorrhages while 7 had extracranial injuries	Children with SDH should be thoroughly examined as SBS is one of the common causes.	
9	Leventhal et al., 2010 [12]	USA	Retrospective	1573	<3 years	Abused children were usually younger and had no insurance.	High incidence of abuse in infancy as compared to another age group especially between 2 and 7 months, before that falls predominate.	
10	Díaz-Olavarriea et al., 2011 [13]	Mexico	Retrospective	13	Mean age: 8 months	Females were the most common victims of AHT and were usually children of young mothers. They have a high incidence of neurological and respiratory compromise.	Identification of risk factors and clinical features is important for timely diagnosis and effective management.	
11	Greiner et al., 2012 [14]	Cincinnati	Retrospective	71	0.5 months to 29 months	The presence of seizures, intubation, and PICU admission was associated with poor CATS, i.e., 35%, 57%, and 34%, and poor CLAMS score (<70) i.e., 32%,52%, and 31%, respectively.	History of seizures or the patients intubated and admitted to PICU are associated with poor development later.	
12	Parks et al., 2012 [15]	United States	Retrospective	780	<5 years	Mortality rates rate /100,000 was, Males: 58%, Age <1 year: 57%, African American: 1.74%	CDC-based definition of AHT can be used for surveillance and thus help to develop preventative strategies.	
13	Hasbani et al., 2013 [16]	Philadelphia	Retrospective	32	<2 years	Discontinuous and slow-disorganized waves on EEG and ischemia on imaging were suggestive of non-convulsive seizures. These patients had more chances of having clinical seizures (67%) and abnormal imaging – parenchymal 61% and extra-axial 56%.	Electrographic seizures and electrographic status epilepticus are common in AHT victims and paralytic agents as well as anticonvulsants do not prevent them.	
14	Babbitt et al., 2013 [17]	United States	Retrospective	28	<3 years	The average presenting glucose was higher for AHT compared with accidental injury (190 vs 133 mg/dL, p<0.001). Patients with AHT had greater PICU and hospital length of stay and more severe disabilities on discharge (p<0.001).	Patients having abnormal neurological examination should be tested for blood glucose and if >140mg/dl should undergo imaging.	
15	Parrish et al., 2013 [18]	Alaska	Retrospective	45	<2 years	Children born to young mothers <20 years of age, unmarried mothers, and mothers with < 12 years of education had significantly higher incidences of AHT.	A thorough history both clinical and sociodemographic of parents is needed in any child with head abuse to rule out abuse.	
16	Niederkrotenthaler et al., 2013 [19]	United States	Retrospective	7603	<2 years	AHT is frequently diagnosed at children’s hospitals (aOR = 1.20). Admissions were more in winter), and on an OPD basis (aOR=1.35). More common among low-income families and Medicaid insurance.	Abused children have higher mortality and hospital stays as compared to NAHT.	
17	Kadom et al., 2013 [20]	Washington, DC, USA	Retrospective	38	Mean age: 164 days	Hypoxic brain injuries were significantly seen in patients of AHT and 81% of such patients had cervical injuries too.	Cervical spine MRI though cannot distinguish between AHT and NAHT. But cervical spine injuries are more common in AHT victims as well as hypoxic brain injuries thus MRI of the brain and cervical spine should be performed in AHT patients.	
18	Bradford et al., 2013 [21]	Pennsylvania	Retrospective	105	<2 years	Homogeneous hyperdense abnormalities on CT disappear within 2-40 days, while mixed lesions between 1-181 days. 5 patterns of SDH observed on MRI.	SDH is common and the abnormality can be seen on imaging within hours. However, the findings could promptly define the time period of injury.	
19	Roach et al., 2014 [22]	Colorado	Retrospective	580	<2 years	AHT patients had more SDH and DAI, while accident cases had more skull fractures and EDH.	They are associated with a high ISS score (22) and also with high rates of mortality so any child with SDH /DAI, always rules out AHT.	
20	Choudhary et al., 2014 [23]	Wilmington, USA	Retrospective	67	<4 years	Cervical spine ligamentous injuries were present in 78% of the AHT patients and they were associated with brain ischemia.	MRI whole spine is preferred in AHT children as difficulty in breathing and hypoxic brain injury can occur due to upper occipitocervical cord injury.	
21	Feldman et al., 2015 [24]	USA	Retrospective	383	NA	Acute on chronic SDH AHT children had less serious injuries; however, the incidence of retinal hemorrhages was the same in both acute as well acute on chronic, i.e., 65% and almost 50% of these patients presented with asymptomatic macrocephaly.	AHT victims having acute or chronic SDH have the unique feature of having asymptomatic macrocephaly and the etiology is mainly new trauma rather than rebleed.	
22	Westrick et al., 2015 [25]	Vanderbilt, USA	Retrospective	139	Median age: 5 months	Mortality (18%) was more common in non-alert and young age (median age: 8.6 months). Patients having hyperglycemia (140 mg/dL) were also associated with high mortality (p<0.0001).	Follow-up of the patients is very important to evaluate the morbidities in these patients and for future prevention programs.	
23	Burkhart et al., 2015 [26]	Houston, Texas	Retrospective	103	<3 years	Children presenting with lethargy or altered mental status (p<0.0001), and subdural hemorrhage (p<0.0001), were likely to have RH. However, the presence of fractures in both the skull and other sites with intracranial hemorrhages was not associated with RH.	RH in AHT is associated with SDH and not with skull fractures.	
24	Tung et al., 2015 [27]	Providence, Rhode Island	Retrospective	9	Mean age: 6.8months	Mixed-density subdural hematoma presented in 67% of cases of NAHT, while homogeneous was seen in 33% of cases.	Mixed-density subdural hematoma is more common in NAHT but not specific as also seen in accidental cases within 48 hours.	
25	Buttram et al., 2015 [28]	Phoenix	Retrospective	37	0-21 years	MRI detected intraparenchymal lesions in 43% as compared to lesions with CT ((11%) which was significant (p=0.03). It was able to detect abnormalities in 75% of patients (6/8) with normal CT scans.	MRI detects more parenchymal lesions than CT with better anatomic details however its unavailability, longer time taken, and sedation are potential problems.	
26	Vadivelu et al., 2016 [29]	United States	Retrospective	28	NA	39% of patients of NAHT, with poor GCS dilatation, occurred within 3 days.	PTV developed early after TBI both accidental and abusive, and it is more commonly seen in SAH patients or those who underwent decompressive craniotomy. However, only a few required shunt surgeries for hydrocephalus in abusive patients.	
27	Khan et al., 2017 [30]	Memphis	Retrospective	282 (28%) had one or more CVA	1–6 months	Stroke in AHT patients was usually bilateral (78%), multifocal (85%), and associated with an overlying subdural hematoma (86%).	Strokes in AHT represent severe injury in terms of high rates of neurosurgical interventions, increased mortality, and readmission.	
28	Gencturk et al., 2017 [31]	Minnesota	Retrospective	16	Mean age: 16.46 months	31% had rib fractures of which 80% were bilateral.	Neuroimaging findings like retinal hemorrhages on SWI and Hypoxic ischemic Injury on MRI help to differentiate AHT from NAHT.	
29	Kralik et al., 2017 [32]	Indiana	Prospective	24	Median patient age: 4 months	Ultrafast MR imaging was 50% sensitive in detecting traumatic pathology while CT had only 25%, and sensitivity increases to 60% when the two are combined.	Ultrafast MRI requires no sedation and is less time-consuming as compared to standard MRI however has low sensitivity.	
30	Morgan et al., 2018 [33]	Omaha, NE, USA	Retrospective	30	<4 years	Retinal hemorrhages and vitreous hemorrhages were commonly seen in AHT patients i.e., 63% and 37%, respectively. SDH was seen in 97% of cases.	Retinal hemorrhages are not specific for AHT but they along with SDH and LOC are highly significant for AHT.	
31	Dingman et al., 2018 [34]	Colorado	Retrospective	58	<5 years	Electrographic seizures 51.2% and HIE 77.4% were common. Correlation of HIE with seizure burden with r=0.61.	HII is more common in AHT features as restricted diffusion on MRI and is highly correlated with the presence of seizures. Early posttraumatic seizures can later lead to epilepsy.	
32	Kahn et al., 2018 [35]	Paris, France	Prospective	224	<3 years	Intraocular hemorrhages were seen in 77.5% of cases of nonaccidental head trauma.	Intraocular hemorrhages when associated with SDH are highly associated with shaken baby syndromes. Also, they do not have specific patterns, which are particular for SBS.	
33	Ronning et al., 2018 [36]	Minnesota	Retrospective	55	<1 year	81.2% of patients with AHT had parasagittal vertex clots and there were 8 times the risk of AHT. They also had a high incidence of no known mechanism of injury i.e., 69.1%, retinal hemorrhage i.e., 75%, and hypoxic-ischemic changes, i.e., 25%.	In the presence of risk factors, parasagittal vertex clots could be suspicious for AHT and warrant further investigations.	
34	Orru et al., 2018 [37]	Baltimore	Retrospective	57	< 5years	HII presented as asymmetric cortical distribution in 66.7% of cases, and diffused lesion in the rest of the cases. It was not significantly correlated with SDH or skull fractures with p=0.6 and 0.53 respectively.	HII presenting as asymmetric cortical distribution in the majority of cases was the most severe form of parenchymal damage in AHT patients and was associated with poor outcomes.	
35	Babl et al., 2018 [38]	Australia and New Zealand	Prospective	372	0-18 years	Intentional injuries are seen in less than 2% of cases of head injury patients with caregivers being the most common perpetrators (27.7%) and they mostly injured young kids <2 years. CTs were also more abnormal in these cases i.e., 47.6%.	Parents are the most common cause of severe injuries in AHT victims.	
36	Henry et al., 2018 [39]	Philadelphia	Retrospective	233	<1 year	Among spine injuries, 23.2% were extra-axial spinal hemorrhage and 8.7% were ligamentous injuries. Also, there was an association between abnormal GCS and cervical spine injuries (p=0.03) as well as moderate to severe head injuries (head MAIS score ≥ 3) and cervical spine injuries (p=0.02).	Cervical spine injuries are associated with moderate to severe head injury and MRI is the preferred modality as ligamentous injuries are more common.	
37	Kralik et al., 2018 [40]	Texas	Retrospective	34	Median patient age was 4 months	BB demonstrated 83% sensitivity 100% specificity & PPV (95%[CI] 46–100%), for diagnosis of a skull fracture.	Black bone MRI sequence is highly sensitive and specific for the detection of skull fractures in AHT.	
38	Oh et al., 2019 [41]	Atlanta	Retrospective	278	<3 years	Altered mental status was associated with nonconvulsive seizures. These patients had longer hospital stays and required rehabilitation.	AHT patients present with Nonconvulsive seizures and nonconvulsive status epilepticus and they are associated with unfavourable outcomes.	
39	Babl et al., 2019 [42]	J Paediatr Child Health	Prospective	23	Mean age: 4.17 years, Median age: 1.4 years	Glasgow coma scale ≤12 had an OR of 30.3, seizures had an OR of 12, and abnormal CT had an OR of 38.3 for AHT patients.	Patients presenting with seizures, loss of consciousness, and especially with parents neglecting any mechanism of history have high rates of abnormal CT so ED clinicians should be vigilant for pediatric abuse.	
40	Emrick et al., 2019 [43]	West Virginia	Retrospective	120	<2 years	The average annual incidence was 21.6/100,000 live births. High rate of fatalities, i.e., 19.2%, and inconsistent history in 100% of cases.	Drug abuse and economic factors lead to a rise in the incidence of AHT and preventative programs are needed.	
41	Lovett et al., 2019 [44]	Columbus	Retrospective analysis of prospective data	15	1 day to 17 years	There was no significant difference in VMCA for children with AHT versus those with non-AHT (p=0.9265) nor in the pulsatility index of the MCA between groups (p=0.2430). Basilar artery flow was not different between groups (p=0.6245).	Transcranial Doppler parameters like MCA velocity and extreme CBFV Children show significant changes as compared to patients with non-AHT. Thus cerebral autoregulation is intact in both cases.	
42	Wright et al., 2019 [45]	Seattle, WA, USA	Retrospective	143	<3 years	Fifty-four of 85 reimaged children (63.5%) with AHT-SDH rebled.	Subdural rebleeding is common within 1^st^ year and occurs in patients who have brain atrophy, ventriculomegaly, macrocephaly, and deep SDHs.	
43	Thamburaj et al., 2019 [46]	Hershey, USA	Retrospective	26	Mean age: 9.1 months	SWI had specificity and a positive predictive value of 100% for detecting retinal hemorrhages though sensitivity is 50%.	Diagnosis of retinal hemorrhages by SWI can be helpful in diagnosing AHT, especially if we have other associated abnormal neuroimaging.	
44	Sidpra et al., 2020 [47]	London	Retrospective	17	Mean age: 137 days	Among skull fractures simple linear fractures most commonly involved parietal bones followed by occipital bones.	The presence of anatomical variants can make it difficult to diagnose skull fractures and physicians must be aware of it.	
45	Burns et al.,2020 [48]	Florida	Retrospective	43	<3 years	Rib fractures (p<0.001), long bone fractures (p<60.001), retinal hemorrhages (p<0.001), seizures (p<0.001), apnoea (p<0.001), and younger than 6 months (p<0.001) were seen.	No mechanism of injury was reported by the caregiver, and seizures and apnoea are associated with unfavorable outcomes.	
46	Even et al., 2020 [49]	Pittsburgh	Retrospective	75	Median age: 4 months	47.3% of AHT patients had anemia and higher ICH volume, i.e., 33.3 mL, than non-inflicted TBI (p<0.001).	Aaemia and an increased ICH volume are more common in AHT and the two are associated with unfavorable outcomes.	
47	Thiblin et al., 2020 [50]	Sweden	Retrospective	36	<1 year	In 30 cases, there were no findings of SDH or extracranial injuries. In the majority of cases, the father was the perpetrator, i.e.,21, followed by the mother and stranger,5 cases each.	Acute SDH or RH are not isolated associated with AHT; however, if risk factors present for abuse then the two are highly suggestive of AHT.	
48	Sayrs et al., 2020 [51]	Phoenix	Retrospective	240	<1 year	64.4% of patients in families with reported intimate partner violence were <12 months of age. IPV was associated with a twofold increase in the risk of AHT.	Violent patterns in the family should be identified to protect minors from abuse.	
49	Rebbe et al., 2020 [52]	Washington	Retrospective	354	<5 years	The strongest risk factor was a prior CPS allegation as >20% have prior CPR reports. 10.5% deaths among admitted patients with 86.5% of those dying within two weeks of admission.	AHT is more common in infancy with maximum cases at the 2^nd^ and 8^th^ month of life. Prior CPS report has an overall hazard ratio of 4 times for hospitalization.	
50	Hymel et al., 2020 [53]	Netherlands	Prospective	160	NA	Caregivers’ denial of history had a specificity of 0.90, PPV of 0.81, and positive LR of 4.83 for abusive head trauma.	No mechanism of injury or changing history by caregivers or inconsistent accidental history are highly specific	
51	Henry et al., 2020 [54]	Philadelphia	retrospective, stratified, random systematic sample	169	<1 year	Encephalomalacia was the most common finding, present in 3.3% of cases, followed by non-parenchymal intracranial hemorrhages in 2.5 % of cases, skull fractures in 0.7%, and cerebral edema in 0.4 % of cases.	With high incidence of neurological abnormalities, neuroimaging should be done in all cases of child abuse.	
52	Rosenfeld et al., 2020 [55]	United States	Retrospective	9482	<1 year	Traumatic brain injuries were most common among non-accidental injuries and had concomitant spinal injuries (86%) and skull fractures (68%), thoracic injuries (57%), and solid organ injuries (38%).	Non-accidental trauma is a burden on the healthcare system and is responsible for polytrauma and high mortality rates, thus a need to implement preventative strategies.	
53	Rabbitt et al., 2020 [56]	Wisconsin	Retrospective	47	<5 years	High incidence of spinal injury in AHT population i.e., 62%. Spine injury was associated with longer ICU stays (p<0.001), lower initial mental status (p=0.01), and longer ventilation times (p=0.001)).	Spine injury is associated with brain injury in the AHT population and has poor outcomes in terms of increased ICU stay and poor GCS so imaging of the spine should be done in such patients.	
54	Lee et al., 2020 [57]	Taiwan	Retrospective	78	<1 year	Predictors of mortality, Initial GCS: OR=0.528 (0.389–0.716) p<0.001 Nadir HB (g/dL): OR=0.6 (0.415–0.867) 0.007, Rotterdam CT score: OR=4.493 (2.102–9.603) <0.01.	Low GCS score, low nadir HB, and higher Rotterdam CT score are predictors of in-hospital mortality, while low Hb (<9.35) is associated with poor neurological outcomes.	
55	Notrica et al., 2021 [58]	Phoenix	Retrospective	242	<5 years	Almost 40% of patients with AHT did not report a method of injury, while fall was the most common mechanism in reported cases. Families of AHT have a history of prior CPS contact (p=0.001), police involvement (p=0.001), substance use (p=0.001), and diagnosis/treatment for mental illness (p=0.036).	AHT surveillance is needed to detect mild forms also as they are responsible for the majority of deaths among head injury children less than 5 years.	
56	Kriss et al., 2021 [59]	Louisville	Retrospective	47	<3 years	In AHT, skull fractures most commonly involved the lambdoid suture (43%; p<0.04), followed by the sagittal (23%), coronal (21%), temporal-squamous (12%), and metopic (1%) sutures.	Skull fracture involving >2 cranial sutures is more commonly associated with AHT than accidental.	
57	Manfield et al., 2021 [60]	Australia	Retrospective	55	<4 years	Long-term follow-up showed 81.8% disability in AHT victims with 53% having behavioral problems, 44% with vision impairment, and 26% having fine motor difficulties and gross motor problems.	Good recovery at the initial stages could also lead to long-term disability; thus, future follow-up is necessary for management and to decrease further morbidities.	
58	Theodorou et al., 2021 [61]	Sacramento, CA, USA	Retrospective	7152	<2 years	9.8 % mortality among AHT victims with higher LOS (5.7 vs 1.6 days, p<0.0001) and higher hospital charges ($34,314 vs $19,360, p<0.001) than children with TBI due to MVC were seen.	AHT victims are a vulnerable population with high odds of mortality and longer hospital stays.	

Discussion

Risk Factors

As seen in the results section, many risk variables have been discovered for AHT. The risk factors are divided into four categories: offender history and type, paternal features, child characteristics, and other/admission characteristics.

Offender/Perpetrator History

According to Hymel et al. [53], the history provided by caregivers that lacks the description of the mechanism of injury has a specificity of for non-incidental head trauma of 90%, giving a specificity of 0.9 for the history. One study reported a change of history by the caregivers [4]. According to a study by Thiblin et al. [50], Sills et al. [9], and Babl et al. [42], the perpetrator is usually the patient's one parent, as observed in 81.82%, 28%, and 27.7% of cases, respectively, followed by peers (26.1%), siblings (12.6%), strangers (9.4%), and persons with no known relation to the child (5.6%). In contrast, Westrick et al. [25] reported that siblings are the perpetrators in 57.6% of cases. According to Notrica et al. [58], 45% of instances are caused by an unknown culprit. Babl et al. [42] found that injuries caused by parents are more common in children under the age of two years (75.7%).

Paternal Factors

According to Parrish et al. [18] in 2013, low maternal age, less than 20 years, is associated with a higher risk of AHT, with an AHT rate of 3.6 times as compared to mothers aged 20-29 years and 20 times as compared to mothers aged > 30 years. Starling et al. [7] reported an odds ratio (OR) of 0.9. Similar findings were also reported by Diaz-Olavarria et al. [13] and Rebbe et al. [52]. Many studies have also observed that children born to unmarried mothers are at higher risk, with Starling et al. [7] and Keenan et al. [5,6] observing an OR of 1.6 and 2.2, respectively. At the same time, Notrica et al. [58] found that married mothers carry a 24% risk. Children of mothers with low educational levels are also at risk. Unintended pregnancies are linked to a higher risk, according to Diaz-Olavarria et al. [13], observing an incidence of 85%; these females also have fewer prenatal visits (<5), further increasing the risk. Starling et al. [7] found that prenatal care initiated after the first trimester has an OR of 1.1, while Keenan et al. [5,6] observed an OR of 2.7. Similarly, Starling et al. [7] reported an OR of 2.2 for AHT if the index child is the first child, while Keenan et al. [5,6] reported an OR of 2.6.

According to Hymel et al. [53], a parent's history of drug misuse increases the risk by 3.51 times. The father's history of alcohol consumption is particularly related to AHT, with a prevalence of roughly 77%. Surprisingly, military parents are also more likely to abuse their children. According to Starling et al. [7], fathers who stay at home have an OR of 0.4 and 0.5, according to Keenan et al. [5,6]. According to Notrica et al. [58], Talvik et al. [10], and Sayrs et al. [51], violence between partners is associated with a rise in AHT, with Sayrs et al. finding an association of twofold.

Patient factors, such as young age (i.e., four months to two years), are linked to an increased risk of AHT. Many studies have found that male children are at higher risk, with Niederkrotenthaler et al. [19] reporting a 60.3% incidence, though Diaz-Olavarria et al. [13] reported a higher incidence in girls. Multiple births and premature births are also linked to an increased risk. Caucasians and whites are at higher risk, with Westrick et al. [25] reporting 63.3% Caucasians and Notrica et al. [58] reporting 37.3% Caucasians. Whites are at higher risk, according to Rebbe et al. [52], with an incidence of roughly 59.3%.

Other Characteristics

According to Feldman et al. [24], AHT is more commonly identified in children admitted to hospitals, with an estimated risk of 1.97, and the risk rises with the number of prior admissions. Parrish et al. [18] reported an increased risk of AHT in patients admitted to a secondary hospital, with a 4.4-fold increase. Westrick et al. [25] reported an OR of 3.3 in rural settings, while Starling et al. [7] reported an OR of 0.5 in urban settings. Winter and autumn are the most common seasons of presentation. Some studies have also mentioned that children on Medicaid and Children’s Health Insurance Program (CHIP) have a higher risk of AHT.

Clinical Features

Patients present with a variety of clinical symptoms. While a mechanism of injury such as a fall, trauma, or motor vehicle accident may be claimed, an inadequate history may lead to suspecting abusive trauma.

AHT patients frequently present with loss of consciousness, disorientation, irritability, amnesia, vomiting, seizures, lethargy, respiratory distress, apnea, and scalp hematoma. When comparing clinical features in children under 12 months, males, those with SDH, or the absence of external signs of injury, AHT should be considered [6-8,11,21,22,24,33].

Seizures are a significant indicator of AHT, with an overall incidence of 20%-50% and a prevalence of 60% in children aged less than one year and 43% in children aged more than one year. Seizures may occur immediately during the incident and, in some cases, across a lifetime. This may be secondary to direct impact, acute change to anatomy, and injury on a cellular/subcellular level. Depending on the patient's age and presentation, the type of seizure may differ. Neonates are more likely to have focal clonic seizures, while neonates with AHT are more likely to have nonconvulsive seizures. Continuous EEG (cEEG) is indicated in such patients since there is a significant risk of recurrence [10,14,16,34,41,42].

Subdural hematoma (SDH) is the most frequent type of brain injury identified in AHT patients, accounting for 46.1%-80.4% of cases. Shaking is considered the most common AHT mechanism. SDH is linked to convulsions, retinal hemorrhages, and bruising on the head and neck. The incidence was comparable in children under and over one year of age. Epidural hemorrhage (EDH) has a lesser frequency of 4.6%. EDH is connected with skull fractures and has been seen in 60%. SDH is related chiefly to shaking, but EDH is related to direct trauma. Other brain injuries reported in AHT patients include diffuse axonal injury (DAI), which is the most prevalent and can only be diagnosed by MRI. Subarachnoid hemorrhage (SAH) is less prevalent in children under one year of age. Hypoxic-ischemic encephalopathy is another significant finding in AHT, and it is most found in conjunction with spinal injuries, while DAI is discovered in 55% of cases. A child with SDH and posterior parenchymal injury had an 80% likelihood of being diagnosed with AHT [6-8,11,21,22,24,33].

Extracranial Injuries

Intraocular injuries: Intraocular hemorrhages are commonly observed in AHT patients. An intraocular hemorrhage is present in 46% of cases. Intracranial injuries, particularly SDH, are frequently associated with these injuries. Retinal hemorrhage is a key indicator and is commonly found in AHT patients. A retinal hemorrhage is present in 28.2%-37% of cases. It is generally bilateral and more common in children under the age of two. The absence of retinal hemorrhage does not rule out AHT, but its presence, especially when bilateral, strongly suggests abuse [6-8,11,21,22,24,33,35].

Spinal injuries: Spinal injuries are common in AHT and should be assessed in all cases. Ligamentous injuries, particularly at the corticomedullary junction, are frequently observed. The spinal injuries are present in 34% of AHT cases, with the cervical spine being the most affected area. MRI is essential for detecting these injuries, which are often missed on CT scans [6-8,32].

Rib fractures and long bone fractures: Rib fractures are commonly associated with AHT. They are often multiple and located posteriorly. The rib fractures are present in 28.1% of cases. Long bone fractures, particularly metaphyseal fractures, are also common. The long bone fractures are present in 22% of AHT cases. These fractures are highly specific for non-accidental trauma, particularly when found in children under the age of one year [48].

Abdominal injuries: Abdominal injuries, particularly liver injuries, are also observed in AHT cases. A study found that liver injuries were present in 15% of cases. These injuries can be severe and are often associated with other signs of abuse, such as bruising and fracture of vertebrae [1-6].

Imaging

Computerized tomography scan: CT scans are essential for the initial assessment of AHT patients. They are effective in detecting skull fractures, intracranial hemorrhages, and brain edema. SDH is the most common finding, with a prevalence of up to 80%. CT scans are particularly useful for detecting acute hemorrhages and skull fractures. The CT scans have a sensitivity of 97% for detecting intracranial injuries. Mixed-density SDH is often seen in AHT and is associated with repeated episodes of abuse. Timing is crucial, and abnormalities can be detected within 72 hours of the injury [42].

Magnetic resonance imaging: MRI is more sensitive than CT for detecting brain injuries in AHT patients. It is particularly useful for detecting hypoxic-ischemic injury, DAI, and chronic SDH. MRI is also valuable for follow-up and detecting rebleeding and other associated abnormalities. Recent advancements, such as ultrafast MRI and black bone MRI, have improved the detection of brain injuries without the need for sedation. MRI is essential for a comprehensive assessment of AHT patients, particularly for detecting subtle injuries that may be missed on CT scans [39,40].

Outcomes

The outcomes of AHT depend on multiple factors, including the severity of the injury, the presence of risk factors, clinical presentation, and imaging findings. Poor outcomes are associated with no mechanism history, seizures, loss of consciousness, apnea, anemia, and a low Glasgow Coma Scale (GCS) score. Long-term outcomes include developmental delays, psychomotor delays, visual abnormalities, and seizures. 68% of AHT survivors had significant developmental delays, while another found that 50% had psychomotor delays. Visual abnormalities, including blindness, are common, with a prevalence of 30%. Seizures are also a significant long-term outcome, with a 20%-50% prevalence [6-8,11,21,22,24,33].

Various tools, such as the Glasgow Outcome Scale-Extended Paediatric version (GOS-E Peds), the Children's Assessment Tool (CAT), and the Child Life and Activity Monitoring System (CLAMS), are used to assess morbidity and long-term outcomes in AHT patients. These tools highlight the significant long-term disabilities that AHT victims may face [14].

AHT is a leading cause of death in pediatric trauma, particularly in children under one year of age. Mortality rates are high, 20%-25%. Multiple factors, including the injury's severity, extracranial injuries, and the timeliness of intervention, influence the mortality rate [6-8,11,21,22,24,33].

## Conclusions

AHT is a significant public health issue with severe short- and long-term consequences. Identifying and managing AHT requires a comprehensive understanding of the risk factors, clinical features, extracranial injuries, and radiological findings. Early recognition and intervention are crucial to improving outcomes in affected children. This systematic review provides a detailed overview of the various aspects of AHT, aiming to enhance the diagnostic and management skills of healthcare professionals dealing with this challenging condition. Through a better understanding of AHT, we can improve the detection, management, and prevention of this devastating form of child abuse.
